# Oncogenic non-V600 mutations evade the regulatory machinery of RAF including the Cdc37/Hsp90 chaperone and the 14-3-3 scaffold

**DOI:** 10.7150/thno.103958

**Published:** 2025-01-06

**Authors:** Xiaoyu Wan, Jiajun Yap, Junjun Chen, Yifan Li, Regina Faruk, Nazereth Chor Boon Tan, Yiying Ma, Yiting Lim, Karlina Bte Jubri, Jingyi Hu, Jimin Yuan, Ge Zhang, Quan Li, Yoon Sim Yap, Paula Lam, Mei Wang, Nai Yang Fu, Jiancheng Hu

**Affiliations:** 1 The Division of Cellular and Molecular Research, National Cancer Centre Singapore, Singapore General Hospital, 30 Hospital Boulevard, Singapore 168583.; 2 The Cancer and Stem Cell Program, Duke-NUS Medical School, 8 College Road, Singapore 169857.; 3 The Division of Medical Oncology, National Cancer Centre Singapore, 30 Hospital Boulevard, Singapore 168583.; 4 The Oncology Academic Clinical Programme, Duke-NUS Medical School, 8 College Road, Singapore 169857.; 5 Department of Physiology, National University of Singapore, 2 Medical Drive, Singapore 117597.; 6 Cellvec Pte. Ltd. 100 Pasir Panjang Road, Singapore 118518.; 7 ACRF Cancer Biology and Stem Cells Division, The Walter and Eliza Hall Institute of Medical Research, Parkville, VIC 3052, Australia.

**Keywords:** BRAF, RAF family kinases, Cdc37/Hsp90 chaperone, 14-3-3 scaffold, RAS/RAF/MEK/ERK signaling, Oncogenic mutations, Targeted cancer therapy

## Abstract

The Ser/Thr kinase RAF, particularly BRAF isoform is a dominant target of oncogenic mutations and many mutations have been identified in various cancers. However, how these mutations except V600E evade the regulatory machinery of RAF protein and hence trigger its oncogenicity remains unclear.

**Methods:** In this study, we used mutagenesis, peptide affinity assay, immunoprecipitation, immunoblot, and complementary split luciferase assay as well as mouse xenograft tumour model to investigate how the function of RAF is cooperatively regulated by Cdc37/Hsp90 chaperones and 14-3-3 scaffolds and how this regulatory machinery is evaded by prevalent non-V600 mutations.

**Results:** We found that Cdc37/Hsp90 chaperones engaged with mature BRAF proteins promoted together with 14-3-3 scaffolds a switch of BRAF proteins from active open dimers into inactive close monomers. Most non-V600 mutations were enriched on or around the Cdc37/Hsp90-binding segments of BRAF, which impair association of CDc37/Hsp90 chaperones with BRAF and hence trap BRAF in active open conformation favouring dimerization. These BRAF mutants with high dimer propensity sustained a prolonged ERK signaling, and were effectively targeted by RAF dimer breaker plx8394 *in vitro* and *in vivo*. In contrast, CRAF and ARAF existed as immature monomers highly packaged with Cdc37/Hsp90 chaperones, which will be released upon dimerization driven by RAS-GTP binding with their N-terminus as well as 14-3-3 scaffold association with their C-terminus. Mature CRAF and ARAF dimers also sustained a prolonged ERK signaling as non-V600 BRAF mutants by virtue of absence of the C-terminal Cdc37/Hsp90-binding segment.

**Conclusions:** Cdc37/Hsp90 chaperones and 14-3-3 scaffolds cooperatively facilitate the switch of RAF proteins from open active dimers to close inactive monomers. Non-V600 mutations disrupt this regulatory machinery, and trap RAF in dimers, which could be targeted by RAF dimer breakers.

## Introduction

The RAF Ser/Thr kinase is a core component of RAS/RAF/MEK/ERK signaling cascade, which plays a central role in cell biology 1-3. Human RAF includes three isoforms, CRAF (or RAF1), BRAF and ARAF, among which BRAF is a dominant isoform with oncogenic mutations likely by virtue of its high basal activity 4. Recent genomic sequencings have identified over 300 BRAF mutations in various cancers. Although a single point mutation, V600E accounts for over 90% cases, there are a significant number of non-V600E mutations in cancers. To target V600E, three type 1.5 RAF inhibitors (Vemurafenib, Dabrafenib, and Ecorafenib) that bind to an αC-helix-out conformation and one type 2 RAF dimer disruptor (Tovorafenib) that engages with a DFG-out conformation have been developed and applied to clinical cancer treatment 5,6. However, there's no drugs available for targeting non-V600 mutations. Understanding the functional modes of non-V600 mutations and developing precise therapeutics for these mutations will significantly improve anti-RAF cancer therapy.

The regulation of RAF family kinases is complicated, which involves in complex molecular interactions as well as phosphorylation and dephosphorylation 7-9. The polypeptide of RAF family kinases consists of a RAS-binding domain (RBD) at N-terminus that mediates its interaction with RAS-GTP and a putative kinase domain at C-terminus that catalyzes the substrate phosphorylation. Like other kinases, Cdc37/Hsp90 chaperones facilitate proper folding of nascent RAF peptides and also regulates their activity afterwards though underlying molecular mechanism remains ambiguous 10-14. Hsp90 inhibitor engagement dissociates Hsp90 from RAF and induces a rapid degradation of RAF 15,16. Although biochemical and structural studies have shown that Cdc37/Hsp90 chaperones recognize both unfold structures 12,14 and the GXFG (or GXYG) motif in Glycine-rich loop of protein kinases 17 that is conserved cross all RAF isoforms, RAF isoforms have quite differential affinity with this chaperone complex 18, suggesting that there is an isoform-specific regulation for RAF-Cdc37/Hsp90 interaction.

The activity of RAF proteins that packed with Cdc37/Hsp90 chaperones is further fine-tuned by 14-3-3 scaffolds 19-22. All RAF isoforms contain two 14-3-3 binding motifs (RSXpSXP): one in Ser/Thr-rich region between RBD and kinase domain, and the other in C-terminal tail following kinase domain. In quiescent status, one 14-3-3 dimer associates with two 14-3-3-binding motifs of one BRAF molecule and thus stabilizes its inactive close monomeric conformation 23,24. Upon stimulation, this intramolecular association of BRAF with a 14-3-3 dimer will be converted into an intermolecular association with one 14-3-3 dimer binding to C-terminal motifs of two BRAF molecules, which promotes side-to-side BRAF dimerization and subsequent transactivation 25,26. Active open BRAF dimer will then be returned back to inactive close monomeric status through ERK-mediated negative feedback phosphorylation as well as reverse 14-3-3 association 27. However, how BRAF cycles between inactive close monomeric status and active open dimeric status with aid of 14-3-3 scaffolds remains elusive so far. Moreover, whether Cdc37/Hsp90 chaperones that associate with BRAF affect BRAF/14-3-3 interaction or reversely is also unclear. As for CRAF and ARAF, there is even less information about how their activity is regulated coordinately by 14-3-3 scaffolds and Cdc37/Hsp90 chaperones.

Oncogenic mutations of RAF alter its conformation and evade its physiological regulations. As for BRAF mutations, the highly prevalent V600E mutation traps BRAF in an active conformation through forming a salt bridge between Lys507 and Glu600 in kinase domain 28. However, functional modes for most non-V600 mutations remain understudied though they have been classified as three groups according to their deferent catalytic activities 29. In this study, we investigated how the function of RAF proteins, particularly BRAF is fine-tuned by Cdc37/Hsp90 chaperones and 14-3-3 scaffolds and how oncogenic non-V600 mutations evade this regulatory mechanism and hence trigger the oncogenicity of BRAF.

## Results

### The C-terminal tail regulates the stability and activity of BRAF but not CRAF and ARAF

**Figure 1:**
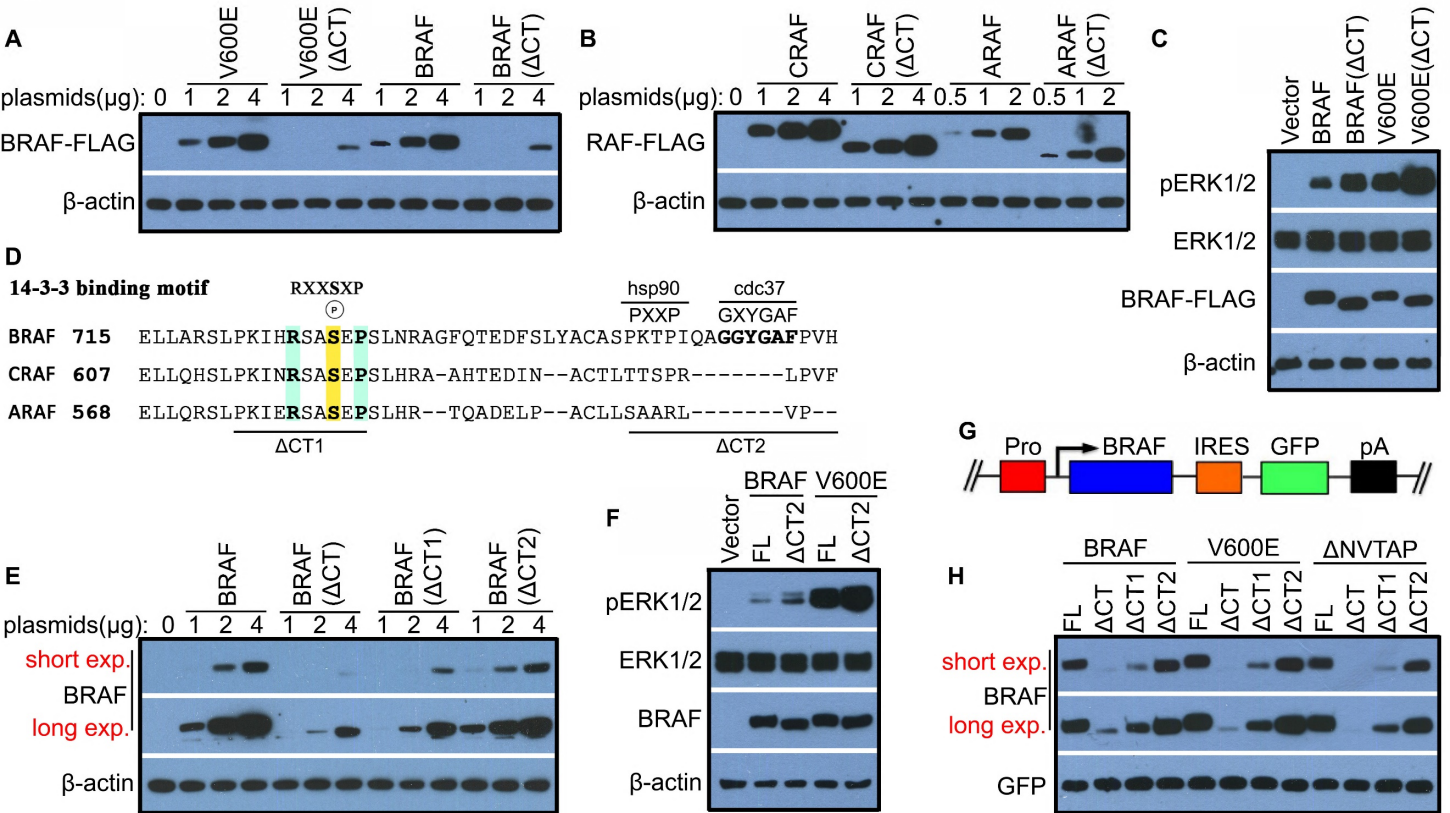
** The C-terminal tail of BRAF but not CRAF or ARAF regulates its stability and activity.** (A) Deleting the C-terminal tail of BRAF or its congenic mutant V600E inhibited protein expression. (B) Deleting the C-terminal tail of CRAF or ARAF did not significantly change their expression. Plasmids with indicated amounts were transfected into 293T cells, and the expression of encoded proteins in 293T transfectants was measured by immunoblots 24 hours after transfections (A and B). (C) BRAF and its oncogenic mutant V600E showed higher activity upon C-terminal tail truncation. Plasmids encoded BRAF, BRAF(V600E), or their truncatants of C-terminal tail were transfected into 293T cells to achieve comparable expression level, and their activities in 293T transfectants were measured by anti-phospho-ERK1/2 immunoblots 24 hours after transfections. (D) Besides a 14-3-3 binding motif (RSXSXP), a potential Cdc37/Hsp90-binding segment (GXYGAF, PXXP) was identified in the C-terminal tail of BRAF but not CRAF or ARAF by sequence alignment. (E) Deleting 14-3-3 binding motif but not Cdc37/Hsp90-binding segment in C-terminal tail significantly impaired BRAF expression. Plasmids encoding BRAF or its mutants were transfected into 293T cells, and protein expression was measured as in (A and B). (F) Deleting Cdc37/Hsp90-binding segment in C-terminal tail significantly elevated the activity of BRAF and BRAF(V600E). Plasmids encoded BRAF, BRAF(V600E), or their truncatants of C-terminal Cdc37/Hsp90-binding segment were transfected into 293T cells to achieve comparable expression level, and their activity in 293T transfectants was measured by anti-phospho-ERK1/2 immunoblots as in (C). (G-H) Validating the roles of C-terminal 14-3-3 binding motif and Cdc37/Hsp90-binding segment for controlling stability of BRAF and its oncogenic mutants. A diagram of BRAF-IRES-GFP expression system for detecting stability of BRAF and its mutants was illustrated in (G). BRAF, V600E, and ΔNVTAP 35-37 encoded by BRAF-IRES-GFP cassette were expressed in 293T cells as in (A), and their expression was measured by immunoblots with GFP as an internal control (H). All images are representative of at least three independent experiments.

Previous studies have shown that the C-terminal tail of RAF family kinases is essential for their function, which mediates 14-3-3 association as well as asymmetric activation 19-22,25,30,31. To further understand underlying molecular basis, here we deleted C-terminal tail of BRAF as well as its oncogenic mutant, V600E and examined their functional alteration in signal transduction. Surprisingly, we found that BRAF and V600E mutant with C-terminal tail truncation were hardly expressed in 293T transfectants in contrast to their prototypes (Figure 1A), which resulted in nearly 4-fold less expression (Figure S1A). However, deleting C-terminal tail of either CRAF or ARAF did not significantly impair their expression in 293T transfectants (Figure 1B and Figure S1B), suggesting that the C-terminal tail differentially regulates the stability of RAF isoforms. We next determined whether the activity of BRAF and V600E is also altered as their stability upon truncation of C-terminal tail, and found that their mutants without C-terminal tail triggered a much stronger ERK signaling when expressed in 293T cells at a comparable level (Figure 1C and Figure S1C). Together, these data suggested that the C-terminal tail plays an important role in controlling the stability and activity of BRAF.

### C-terminal tail regulates the stability and activity of BRAF likely via 14-3-3 scaffolds and Cdc37/Hsp90 chaperones

To understand how the C-terminal tail deletion destabilizes BRAF and enhances its activity, we aligned the C-terminal tail sequence of BRAF, CRAF and ARAF, and found that besides the 14-3-3 binding motif (RSXpSXP) that is conserved across all RAF isoforms, BRAF contained a potential Cdc37/Hsp90 binding segment (GXYG and PXXP) that is overlapped with the aromatic cluster and absent in CRAF and ARAF (Figure 1D) 17,32-34. Hence, we deleted these two fragments of BRAF respectively and measured the stability and activity of two mutants. We found that deleting 14-3-3 binding motif (ΔCT1, Δ_721_PKIHRSASEP_730_) but not Cdc37/Hsp90 binding segment (ΔCT2, Δ_751_PKTPIQAGGYGAFPVH_766_) severely impaired expression of BRAF (Figure 1E), while deleting Cdc37/Hsp90 binding segment significantly elevated activity of BRAF (Figure 1F and Figure S1D). To validate this finding, we next expressed these deletion mutants of BRAF with an IRES (Internal Ribosome Entry Site)-GFP cassette (Figure 1G), which enables us to exclude the potential artifact arising from transfection and transcription by using GFP as an internal control. With this expression system, we confirmed that indeed the 14-3-3 binding motif but not Cdc37/Hsp90 binding segment in C-terminal tail played the major role in controlling the stability of BRAF as well as V600E and β3-αC loop-deletion (Δ_486_NVTAP_490_) mutants 35-37 (Figure 1H). Since previous studies suggested that constitutively active BRAF mutants were less stable than their wild-type counterpart 15,16, we further investigated whether the catalytic activity of BRAF played a role in the stability of BRAF regulated by its C-terminal tail. We thus mutated the catalytic lysine in the VAIK motif of BRAF(V600E) 38, and found that it did not significantly alter the stability of BRAF(V600E) with truncation of C-terminal tail (Figure S1E). Overall, our data clearly demonstrated that the 14-3-3 binding motif and the Cdc37/Hsp90 binding segment in the C-terminal tail of BRAF regulates its stability and activity respectively.

### BRAF contains multiple Cdc37/Hsp90 binding segments that are highly mutated in human cancers

**Figure 2:**
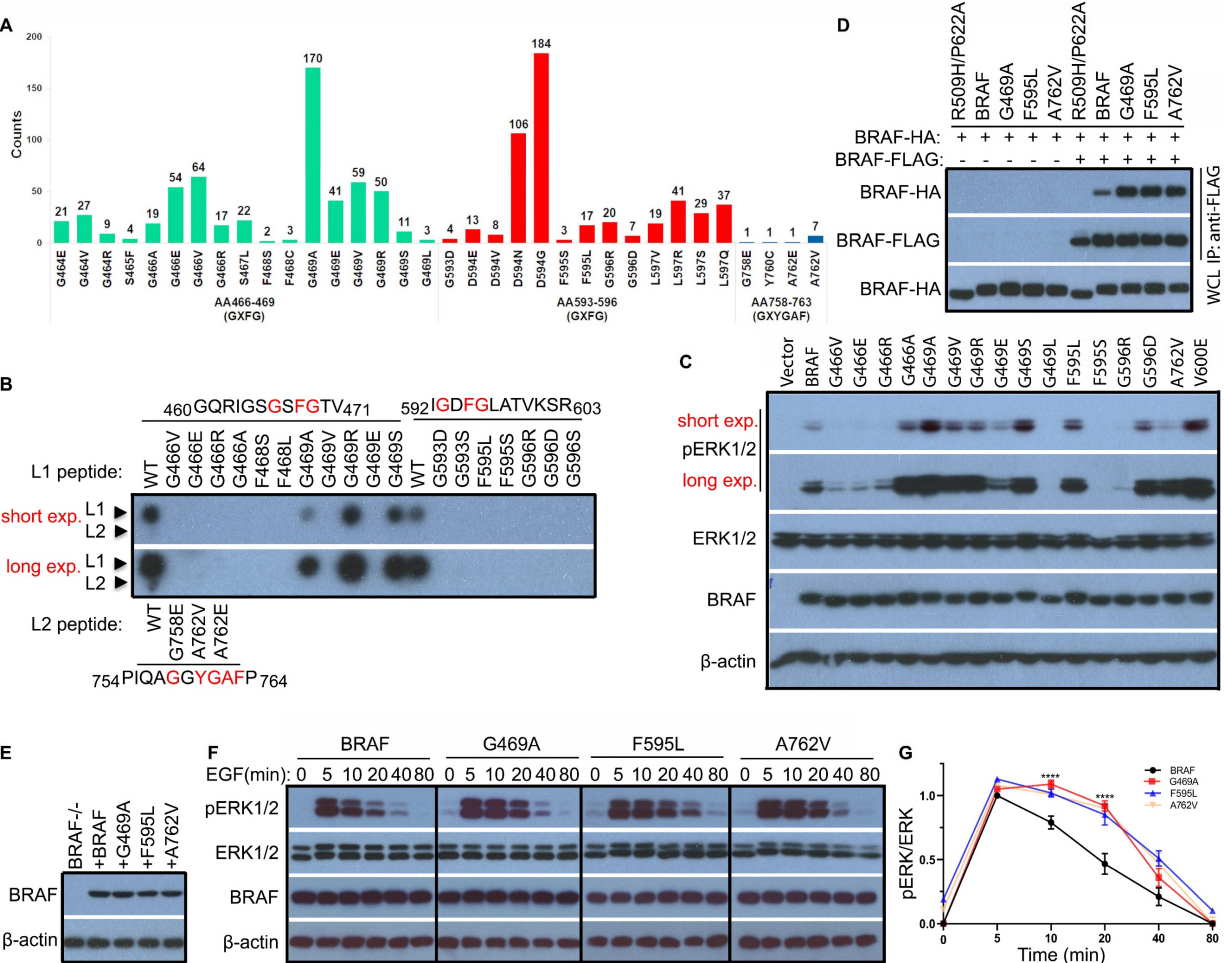
** Prominent non-V600 mutations alter the Cdc37/Hsp90-binding segments of BRAF, which promote BRAF dimerization and enable BRAF to sustain a prolonged ERK signaling.** (A) Most prominent non-V600 mutations in cancer genomes occured on/around Cdc37/Hsp90 binding segments of BRAF. This data was extracted from the COSMIC database. (B) Mutations on Cdc37/Hsp90 binding segments impaired association of Cdc37 with BRAF. The peptide affinity assay was carried out as described in Methods and Materials. (C and D) BRAF mutants with altered Cdc37/Hsp90-binding segments had different kinase activity but constantly elevated dimer affinity. BRAF mutants were expressed in 293T cells, and their activity was measured by anti-phospho-ERK1/2 immunoblot as above (C). HA-tagged BRAF and its mutants were expressed alone or co-expressed with FLAG-tagged counterparts in 293T cells, and their homodimerization was measured by anti-FLAG immunoprecipitation combined with anti-HA and anti-FLAG immunoblots as described before 37,50,66 (D). (E-G) BRAF mutants with altered Cdc37/Hsp90-binding segments sustained a prolonged ERK signaling induced by EGF. To reconstitution of BRAF-/- fibroblasts with wild-type or BRAF mutants, BRAF-/- fibroblasts were infected by retroviruses encoding BRAF or mutants with IRES-GFP cassette, and the GFP^low^ populations for individual infections were sorted out by flow cytometry. The expression of BRAF or its mutants in these sorted stable cell lines was measured by immunoblot (E). Then stable cell lines in (E) were stimulated with EGF as indicated time, and their ERK activity was measured by anti-phospho-ERK1/2 immunoblot (F) as well as quantified by using Image J (G). All images are representative of at least three independent experiments.

Cdc37/Hsp90 chaperones play a crucial role in the regulation of protein kinase function, which involves in kinase folding, maturation, and activity modulation 39. Recent studies suggested that the Cdc37/Hsp90 complex sensed both specific sequences (such as GAF and GXF(Y)G) and unfold/dynamic conformations of kinase clients 12,17,40. Here we scanned the whole protein sequence of BRAF for potential Cdc37/Hsp90 binding segments and found that there were three segments locating around _466_GSFG_469_ (Gly-rich loop), _593_GDFG_596_ (DFG motif)_,_ and _758_GGYGAF_763_ (C-terminal tail) respectively. Surprisingly, we found that all these Cdc37/Hsp90 binding segments were frequently mutated in cancer genomes and most prominent non-V600 mutations occurred on or around these segments (Figure 2A and Figure S2A). Moreover, all these mutations except G469R significantly impaired the Cdc37 association in a peptide affinity assay though their wild-type counterparts themselves had differential Cdc37 affinity with _466_GSFG_469_ > _593_GDFG_596_ > _758_GGYGAF_763_ (Figure 2B). To validate this finding, we also purified three representative mutants from 293T cell transfectants, and found that they had a slightly less Cdc37/Hsp90 association (Figure S2B). These data suggested that partially disrupting Cdc37/Hsp90 chaperone association potentially triggered the oncogenicity of BRAF. However, when expressed in 293T cells, these BRAF mutants exhibited quite different activities with either up- or down-regulation (Figure 2C and Figure S2C). Since both constitutively active and kinase-impaired RAF mutants could be oncogenic only if they have elevated dimer affinity 37,41-45, we next determined whether these Cdc37/Hsp90-related mutants had altered dimer affinity by using co-immunoprecipitation assay. Indeed, we found that all representative mutations in three Cdc37/Hsp90 binding segments remarkably enhanced BRAF dimer affinity (Figure 2D), suggesting that disrupting Cdc37/Hsp90 association really promotes BRAF dimerization. This conclusion was further strengthened by our finding that a temporary treatment with Hsp90 inhibitor improved BRAF dimerization when expressed in 293T cells (Figure S3).

### BRAF mutants with altered Cdc37/Hsp90-binding segment sustain a prolonged ERK signaling

Physiological ERK signaling is tightly regulated by negative feedback loops, which restrict its amplification and duration, and cancer mutations that enhance RAF dimerization have been shown to sustain an extended ERK signaling 46. Given the elevated dimer affinity of representative BRAF mutants with altered Cdc37/Hsp90 binding segment, we examined their property for transmitting ERK signaling. To reach this goal, we reconstituted BRAF-deficient fibroblasts with either wild-type BRAF or its mutants via retroviral transduction and sorted out the population with equal but also relatively low expression (Figure 2E). We then found that the EGF-triggered ERK signaling had a much slower decay in fibroblasts with representative BRAF mutants than that with wild-type counterpart (Figure 2F-G), which is consistent with a previous report 46. Collectively, our data demonstrated that mutations on Cdc37/Hsp90 binding segments of BRAF altered its property for transmitting RAS signaling, which is related to the enhanced dimerization of BRAF.

### BRAF mutants with altered Cdc37/Hsp90-binding segment can be targeted by type 2 RAF dimer breaker plx8394 but not type 1.5 RAF inhibitor plx4720 *in vitro* and *in vivo*

**Figure 3:**
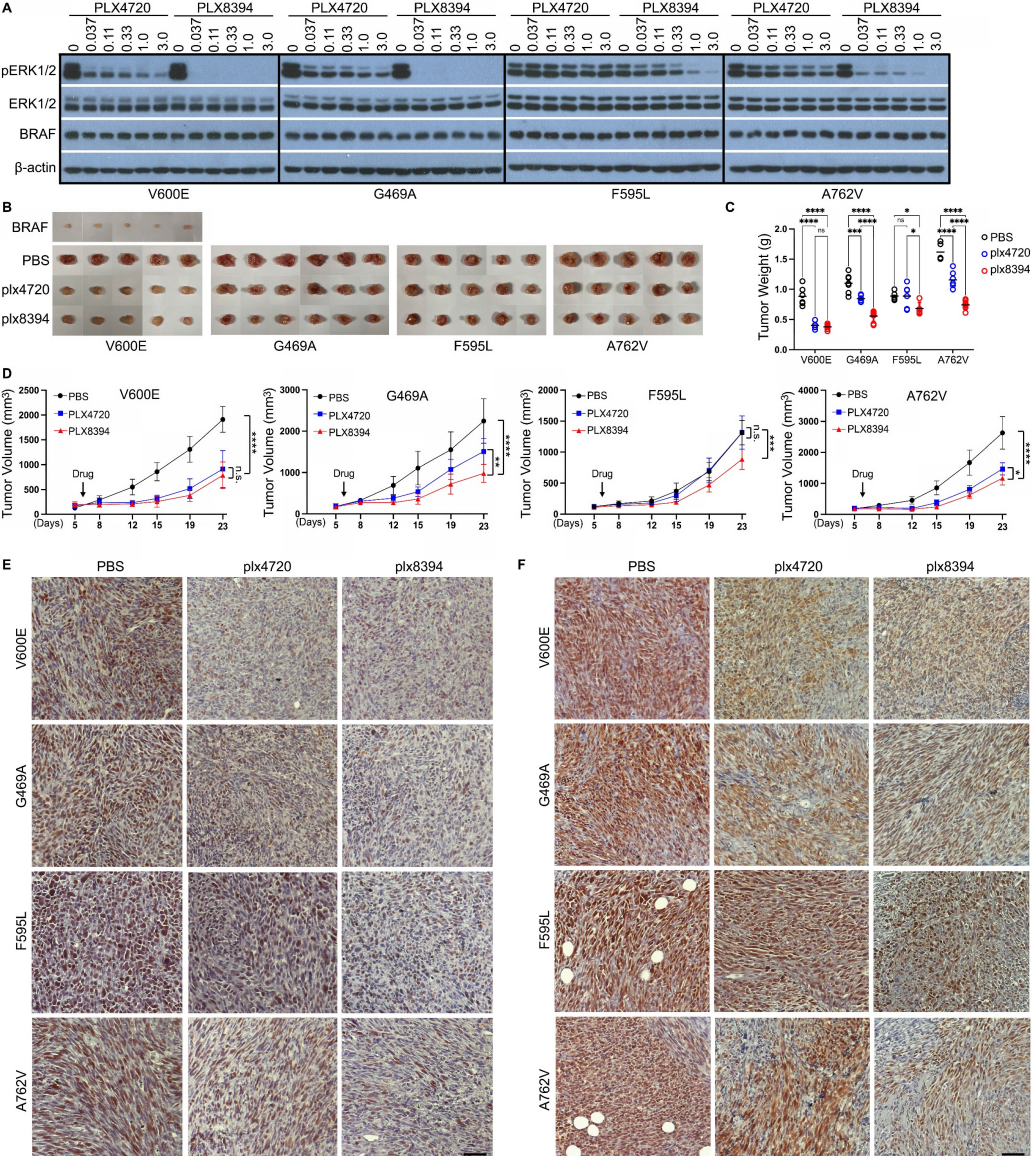
** BRAF mutants with altered Cdc37/Hsp90-binding segments are resistant to type 1.5 RAF inhibitor plx4720 but not to RAF dimer breaker plx8394 *in vitro* and *in vivo*.** (A) The activity of BRAF mutants with altered Cdc37/Hsp90-binding segments was inhibited by RAF dimer breaker plx8394 but not type 1.5 RAF inhibitor plx4720. The 293T transfectants that express different BRAF mutants were treated with RAF inhibitors with indicated concentrations for 90 min, and then lysed for measuring ERK activity by anti-phopho-ERK1/2 immunoblot. (B-F) BRAF mutants with altered Cdc37/Hsp90 binding segments induced fibroblastomas *in vivo*, which can be inhibited by RAF dimer breaker plx8394 but not type 1.5 RAF inhibitor plx4720. The immortalized BRAF-/- fibroblasts were reconstituted with BRAF or its mutants respectively and subcutaneously injected into NOD/SCID mice that were administered orally with PBS, plx4720 or plx8394 respectively. The tumor volume was tracked twice a week, and tumors were harvested at experimental endpoint for immunohistological analysis. The tumor images and weights at experimental endpoint were shown in (B) and (C), while the tumor growth curves with different treatments in (D). The immunohistological stainings of Ki67 and phospo-ERK1/2 in tumor sections were shown in (E) and (F) respectively. Scale=50 µm. All images are representative of at least five mice per group and three independent experiments.

The enhanced dimerization of RAF is also one of major causes that lead to drug resistance in targeted cancer therapy with the clinical RAF inhibitors 47. We thus determined whether these BRAF mutants with altered Cdc37/Hsp90-binding segment were sensitive to plx4720, an experimental version of clinical type 1.5 RAF inhibitor Vemurafenib, or type 2 RAF dimer breaker plx8394. When expressed in 293T cells, these mutants triggered a strong ERK signaling that is much more sensitive to plx8394 than plx4720 (Figure 3A and Figure S4A), suggesting that a DFG-out conformation leads to a stronger catalytic inhibition of BRAF mutants than an αC-helix-out conformation, particularly for BRAF mutants with elevated dimer affinity. As for the partial inhibition of F595L mutant by plx8394, it might arise from altered drug association since the phenol ring of Phe595 directly involves in drug engagement 48.

In order to further confirm the effectiveness of plx8394 to target BRAF mutants with altered Cdc37/Hsp90-binding segment *in vivo*, we next constructed fibroblastoma mouse models by subcutaneously injecting immortalized fibroblasts that were reconstituted with wild-type BRAF or its mutants (V600E, G469A, F595L, and A762V) into NOD-SCID mice respectively. These BRAF mutants induced effectively fibroblastomas *in vivo* in contrast to their wild-type counterpart (Figure 3B), suggesting that all of them are really cancer drivers. Using these models, we demonstrated that all fibroblastomas except that induced by V600E exhibited a strong resistance to plx4720 albeit sensitivity to plx8394 with different extents (Figure 3C-D), suggesting that indeed RAF dimer breakers associating with a DFG-out conformation may serve as a therapeutic option for treating cancer patients harboring these BRAF mutations. To validate this finding, we carried out pathological analysis of these fibroblastomas treated with or without drugs. We found that plx8394 inhibited the expression of Ki67, a tumor proliferation marker, and down-regulated the level of phospho-ERK1/2 significantly in all fibroblastomas while plx4720 had a comparable effect only in V600E-induced fibroblastomas (Figure 3E-F and Figure S4B-C), which supports our opinion.

### 14-3-3 scaffolds regulate association of Cdc37/Hsp90 chaperones with BRAF, CRAF and ARAF

**Figure 4:**
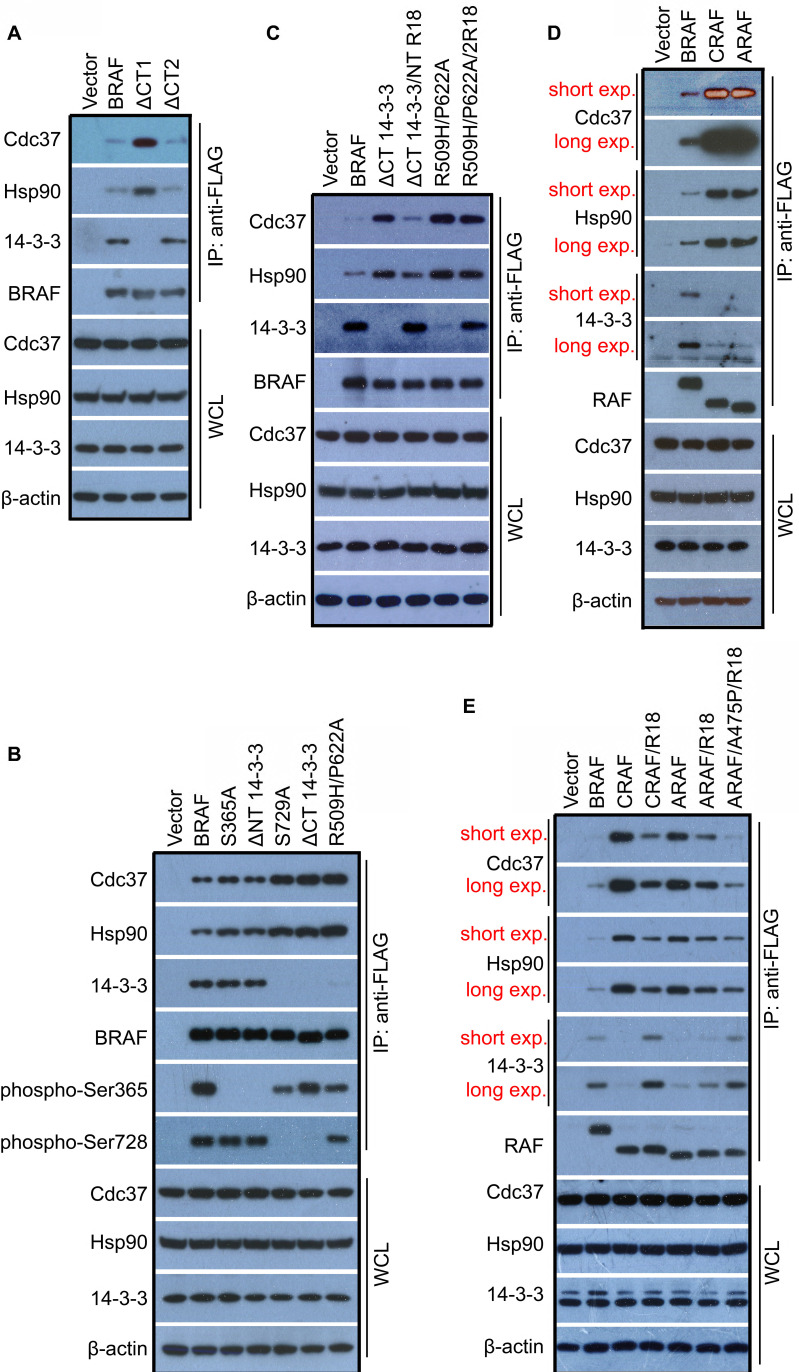
** 14-3-3 scaffold proteins play a critical role in maturation of RAF proteins that facilitated by Cdc37/Hsp90 chaperones.** (A-C) Association of 14-3-3 scaffolds with BRAF proteins triggered a release of Cdc37/Hsp90 chaperones that bound to mature BRAF proteins. Deleting C-terminal 14-3-3 binding motif but not Cdc37/Hsp90-binding segment impaired release of Cdc37/Hsp90 chaperones from BRAF proteins (A). Mutating C-terminal but not N-terminal 14-3-3 binding motif or disrupting dimerization of BRAF inhibited release of Cdc37/Hsp90 chaperones from BRAF proteins (B). Replacing N-terminal 14-3-3 binding motif with R18 segment promoted release of Cdc37/Hsp90 chaperones from BRAF proteins without C-terminal 14-3-3 binding motif but not from monomeric BRAF mutants (C). FLAG-tagged BRAF or its mutants were expressed respectively in 293T cells, and then immunoprecipitated by using anti-FLAG beads. Endogenous Cdc37, Hsp90, and 14-3-3 proteins in immunoprecipitants were detected by immunoblots. (D and E) CRAF and ARAF had much higher loading of Cdc37/Hsp90 chaperones but less association with 14-3-3 scaffolds, which can be reversed by replacing C-terminal 14-3-3 binding motif with R18 segment as together with canonical APE motif. Experiments were carried out as in (A-C) with plasmids encoding FLAG-tagged CRAF, ARAF, or their mutants. All images are representative of at least three independent experiments.

Since Cdc37/Hsp90 chaperone is essential for kinase folding and maturation, and deleting C-terminal 14-3-3 binding motif destabilizes BRAF, we thought that there might be an interplay between Cdc37/Hsp90 chaperone and 14-4-3 scaffold, which affects the folding/maturation of BRAF. To testify this speculation, we detected association of Cdc37/Hsp90 chaperones with BRAF upon deleting C-terminal 14-3-3 binding motif (ΔCT1). Indeed, we found that BRAF(ΔCT1) bound much more Cdc37/Hsp90 chaperones than its wild-type counterpart (Figure 4A, lane 3), suggesting that 14-3-3 association with BRAF is required for its maturation. In contrast, BRAF(ΔCT2) had a similar or slightly less association with Cdc37/Hsp90 chaperones as its wild-type counterpart (Figure 4A, lane 4), showing that C-terminal Cdc37/Hsp90 association itself is dispensable for BRAF maturation. In addition, deleting C-terminal 14-3-3 binding motif completely abolished 14-3-3 association with BRAF even if N-terminal 14-3-3 binding motif maintains intact (Figure 4A, lane 3), suggesting that 14-3-3 scaffolds bind to C-terminal motif first and then to N-terminal motif. This finding illustrated a previous observation that a phospho-peptide derived from the C-terminal 14-3-3-binding motif has a much stronger potency to deplete 14-3-3 proteins from RAF complexes than that generated from the N-terminal 14-3-3-binding motif 22.

To further understand the role of 14-3-3 in the maturation of BRAF, we mutated or deleted N-terminal 14-3-3 binding motif of BRAF, and found that it did not significantly alter Cdc37/Hsp90 association with BRAF as well as overall 14-3-3 association with BRAF (Figure 4B, lane 3-4). In contrast, abolishing C-terminal 14-3-3 association (S729A or ΔCT1) or dimerization (R509H/P622A) led to much heavier loading of Cdc37/Hsp90 chaperones and a complete loss of 14-3-3 association with BRAF even if the N-terminal 14-3-3 binding motif(s) remained phosphorylated (Figure 4B, lane 5-7). To confirm this finding, we replaced the 14-3-3 binding motif(s) with an engineering 14-3-3-binding segment R18(HCVPRDLSWLDLEANMCL) in BRAF(ΔCT1) or monomeric BRAF(R509H/P622A) 37,49,50, and determined whether it altered association of Cdc37/Hsp90 chaperones as well as 14-3-3 scaffolds with these BRAF mutants. When these BRAF mutants were expressed in 293T cells, we found that replacing the N-terminal 14-3-3 binding motif of BRAF(ΔCT1) with R18 segment dramatically enhanced 14-3-3 association and reduced Cdc37/Hsp90 loading. However, although replacing 14-3-3 binding motifs of monomeric BRAF(R509H/P622A) with R18 segments significantly improved its 14-3-3 association, it did not alter its Cdc37/Hsp90 loading (Figure 4C), suggesting that in the process of BRAF maturation, Cdc37/Hsp90-packed BRAF proteins need to form dimer first and then 14-3-3 scaffold incorporation promotes release of Cdc37/Hsp90 chaperones from mature BRAF proteins.

We next investigated whether 14-3-3 scaffolds regulate the association of Cdc37/Hsp90 chaperones with CRAF and ARAF. In contrast to BRAF, CRAF and ARAF had much weaker association with 14-3-3 scaffolds albeit much heavier loading of Cdc37/Hsp90 chaperones when expressed in 293T cells (Figure 4D), which further supports our opinion that 14-3-3 association with mature RAF complex triggers release of Cdc37/Hsp90 chaperones. Furthermore, deleting C-terminal tail of CRAF and ARAF or disrupting their dimerization by mutations did not further enhance Cdc37/Hsp90 loading on CRAF and ARAF as that on BRAF (Figure S5A-B), suggesting that CRAF and ARAF stay in an immature monomeric status. This finding is consistent with previous observations that CRAF and ARAF have much lower dimer affinity and exist mainly as monomers in quiescent cells 28,30,37. As that for BRAF(ΔCT1), if we replaced C-terminal 14-3-3 binding motif of CRAF and ARAF with R18 segment, these two RAF isoforms showed a stronger association with 14-3-3 albeit a little less Cdc37/Hsp90 loading (Figure 4E, lane 4 and 6). Interestingly, if we converted non-canonical AAE motif of ARAF into an APE motif for facilitating ARAF dimerization 37, we found that R18 segment further enhanced 14-3-3 binding and reduced Cdc37/Hsp90 loading on ARAF (Figure 4E, lane 7), which further support that RAF dimerization is critical for 14-3-3 binding and subsequent release of Cdc37/Hsp90 chaperones.

### Cdc37/Hsp90 chaperones and 14-3-3 scaffolds cooperatively turn active dimeric BRAF complex into inactive monomeric status upon maturation

**Figure 5:**
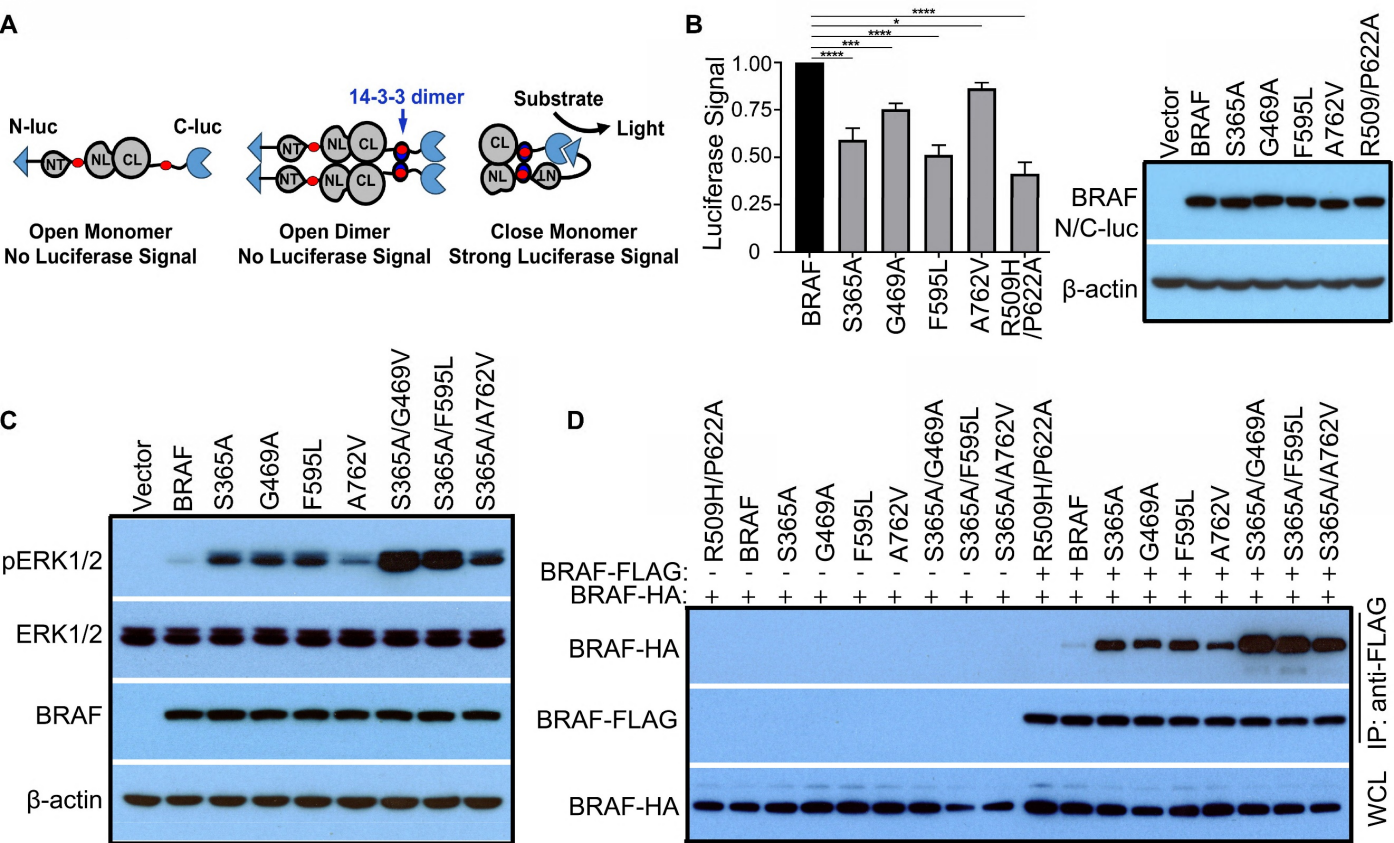
** BRAF cycles between active dimers and inactive monomers, which is fine-tuned by Cdc37/Hsp90 chaperones and 14-3-3 scaffolds.** (A and B) Mutations on N-terminal 14-3-3 binding motif or on Cdc37/Hsp90 binding segments impaired transition of active open conformation to inactive close conformation of mature BRAF. A diagram showed how to use complimentary split luciferase reporter to detect open versus close conformations of BRAF and its mutants (A). Monomeric BRAF mutant (R509H/P662A), N-terminal 14-3-3 binding motif mutant (S365A), and Cdc37/Hsp90-binding segment mutants (G469A, F595L, or A762V) were trapped in an open conformation by different extents (B). N-luc and C-luc were fused to two ends of BRAF and their mutants. Then fusion proteins were expressed respectively in 293T cells, and their luciferase signals were measured as described in Materials and Methods (n=4, *p<0.05, ***p<0.001, ****p<0.0001). (C and D) Mutating N-terminal 14-3-3 binding motif and Cdc37/Hsp90-binding segments had an additive effect to trap BRAF in active dimers. BRAF mutants were expressed in 293T cells, and their activity was measured by anti-phospho-ERK1/2 immunoblot (C). HA-tagged BRAF and its mutants were expressed alone or co-expressed with FLAG-tagged counterparts in 293T cells, and their homodimerization was detected by anti-FLAG immunoprecipitation combined with anti-HA and anti-FLAG immunoblots as described before 37,50,66 (D). All images are representative of at least three independent experiments.

Our data indicated that 14-3-3 scaffold binds to C-terminal tail of BRAF and promotes release of Cdc37/Hsp90 chaperones from mature BRAF complex. However, a significant amount of Cdc37/Hsp90 chaperones still associated with mature BRAF. We speculated that these retained Cdc37/Hsp90 chaperones might facilitate a switch of active dimeric BRAF into inactive monomeric counterpart with a rearrangement of 14-3-3 association. If this is true, disrupting Cdc37/Hsp90 or N-terminal 14-3-3 associations with mature BRAF would block this transition and trap BRAF in an active dimeric status. To justify this notion, we fused a split luciferase to both N-terminus and C-terminus of BRAF or its mutants (N-luc-BRAF-C-luc), which is used as a luciferase reporter to detect a close versus open conformation of BRAF and its mutants (Figure 5A) 50,51. When expressed in 293T cells, we found that wild-type BRAF reporter generated a high luciferase signal (Figure 5B), suggesting that wild-type BRAF exists as a close monomer in quiescent cells as reported before 29,51. In contrast, the luciferase reporter of monomeric BRAF mutant(R509H/P622A) generated a much lower luciferase signal in 293T cells, suggesting that immature monomeric BRAF packing with Cdc37/Hsp90 chaperones has an extended/open conformation. Previous studies have suggested that the close conformation of wild-type BRAF is stabilized by a 14-3-3 dimer that binds to both N-terminus and C-terminus of a same BRAF molecule, and disrupting 14-3-3 association with N-terminus of BRAF will turn BRAF into an open conformation. Indeed, S365A mutation on N-terminal 14-3-3 binding motif of BRAF significantly reduced the luciferase signal of N-luc-BRAF-C-luc reporter, indicating that intramolecular association of BRAF with 14-3-3 dimer is critical for maintaining its close conformation. Using this luciferase reporter approach, we further examined whether Cd37/Hsp90 chaperones regulate transition of mature BRAF from active open conformation to inactive close conformation. We found that mutations on Cdc37/Hsp90-binding segments (G469A, F595L, or A762V) significantly reduced the luciferase signal of N-luc-BRAF-C-luc reporter, suggesting that disrupting Cdc37/Hsp90 association with mature BRAF traps it in an open conformation, which is consistent with a previous report 51. Since both intramolecular 14-3-3 association and Cdc37/Hsp90 engagement with mature BRAF facilitate its transition from active open conformation into inactive close conformation, we next determined whether disrupting both types of interactions had additive effects for triggering BRAF activity as well as its dimerization. As shown in Figure 5C-D and Figure S6, double mutations that simultaneously disrupt both intramolecular 14-3-3 binding and Cdc37/Hsp90 engagement stimulated the activity of BRAF and improved its homodimerization much more potent than single mutations when expressed in 293T cells, suggesting that 14-3-3 scaffolds and Cdc37/Hsp90 chaperones cooperatively trap mature BRAF in an inactive close monomeric conformation.

### The regulatory machinery that comprises Cdc37/Hsp90 chaperones and 14-3-3 scaffolds controls the maturation and activity of not only BRAF but also CRAF and ARAF

**Figure 6:**
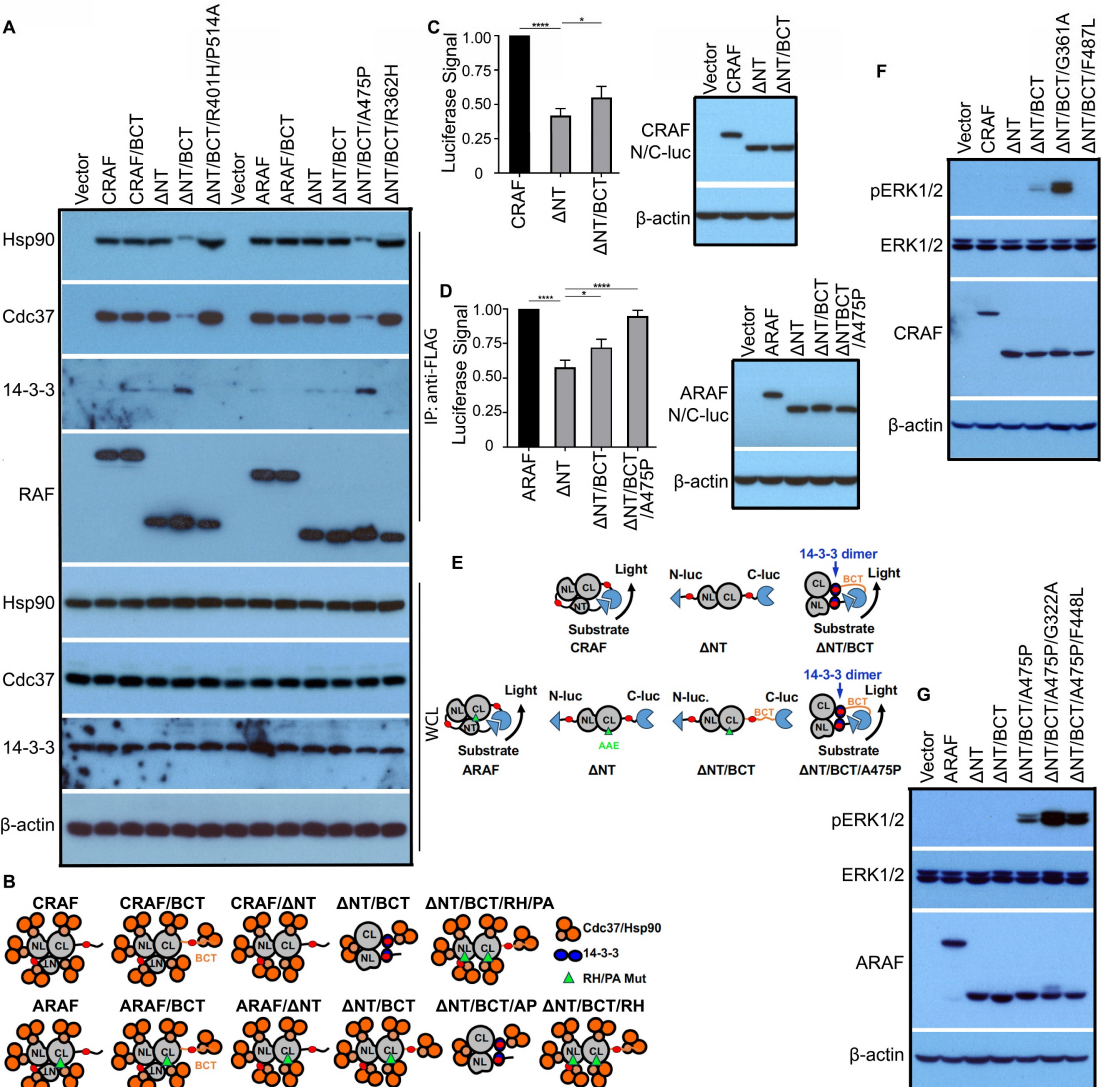
** The maturation and activity of CRAF and ARAF proteins is regulated cooperatively by Cdc37/Hsp90 chaperones and 14-3-3 scaffolds via a manner similar to that of BRAF.** (A and B) Replacing C-terminal tail of CRAF with that of BRAF together with N-terminal truncation improved its maturation, while it still required a canonic APE motif alteration for ARAF maturation. FLAG-tagged CRAF, ARAF, or their mutants were expressed respectively in 293T cells, and immunoprecipitated by using anti-FLAG affinity beads. Endogenous Hsp90, Cdc37, and 14-3-3 proteins in immunoprecipitants were detected by immunoblots as above (A). A diagram to illustrate the potential conformations of CRAF, ARAF and their mutants associating with Cdc37/Hsp90 chaperones and 14-3-3 scaffolds (B). CRAF, CRAF/BCT, and CRAF(ΔNT) had immature conformations with heavier Cdc37/Hsp90 loading, while CRAF(ΔNT/BCT) formed a mature conformation with less Cdc37/Hsp90 association. A double mutation (R401H/P514A) that disrupts dimerization prevented CRAF(ΔNT/BCT) from achieving a mature conformation. As for ARAF, this was more complicated since ARAF has a non-canonic APE motif that is identical to the P514A mutation of CRAF. To achieve a mature conformation, ARAF needed N-terminal deletion, C-terminal replacing and APE motif switching. (C-E) CRAF and ARAF had an immature close monomeric conformation without 14-3-3 association, which can be switched into a mature close monomeric conformation with intramolecular 14-3-3 association through replacing C-terminal tail with that of BRAF and N-terminal truncation (as well as APE motif alteration for ARAF). To probe conformations of CRAF, ARAF and their mutants by complimentary split luciferase assays, N-luc and C-luc were fused to two ends of CRAF, ARAF, and their mutants, which were expressed in 293T cells. Then luciferase assays were carried out as in Figure 5B (n=4, *p<0.05, ****p<0.0001) (C and D). The potential conformations of CRAF, ARAF and their mutants were shown in (E). (F-G) Mutating Cdc37/Hsp90-binding segments of mature CRAF or ARAF mutants triggered their kinase activity. CRAF, ARAF and their mutants were expressed in 293T cells and their activity was measured by anti-phospho-ERK1/2 immunoblots. All images are representative of at least three independent experiments.

Although previous studies had shown that all RAF proteins existed as monomers in quiescent cells 28, here we found that CRAF and ARAF had much tighter Cdc37/Hsp90 association and less 14-3-3 binding in contrast to BRAF, suggesting that unlike BRAF, nascent CRAF and ARAF proteins are immature. To understand molecular mechanism(s) underlying this phenomenon, we firstly replaced their C-terminal tails with that of BRAF, and found that it did not significantly alter their association with Cdc37/Hsp90 chaperones and 14-3-3 scaffolds (Figure 6A, lane 1-3). Since N-terminus of CRAF had been shown to fold back and dock on its kinase domain, and hence inhibit its activity 52, we further deleted N-terminus(aa1-245) of CRAF with replaced C-terminal tail, and found that it dramatically reduced Cdc37/Hsp90 loading and simultaneously enhanced 14-3-3 association, which was diminished by a double mutation(R401H/P514A) that disrupt dimerization (Figure 6A, lane 4-6). This finding suggested that maturation of nascent CRAF protein requires a release of N-terminus from kinase domain as well as Cdc37/Hsp90 chaperones and 14-3-3 association with its C-terminal tail. As for ARAF, only ARAF(Δaa1-202/BCT/A475P) mutant had much less Cdc37/Hsp90 loading and enhanced 14-3-3 association that comparable to mature CRAF mutant (Δaa1-245/BCT) (Figure 6A, lane 7-13), suggesting that besides removing inhibitory N-terminus and replacing C-terminal tail, a canonical APE motif that improves dimerization is also required for its maturation. These potential conformations of CRAF, ARAF and their mutants associating with Cdc37/Hsp90 chaperones and 14-3-3 scaffolds were illustrated in Figure 6B.

We next probed conformational switches of CRAF and ARAF during their maturation by using complementary split luciferase assay. As shown in Figure 6C-E, full-length CRAF and ARAF produced a strong luciferase signal when fused with N-luc and C-luc respectively, suggesting that they stay in a close monomeric status in which N-terminus docks on C-terminal kinase domain. In contrast to full-length counterpart, N-terminal truncatant, CRAF(Δaa1-245) generated much less luciferase signal when fused with N-luc and C-luc, suggesting that immature C-terminal kinase domains of CRAF exists as an open monomer. However, this weak luciferase signal was significantly elevated when its C-terminal tail was replaced with that of BRAF, suggesting that mature C-terminal kinase domain of CRAF with an alternative tail exists as a close monomer like BRAF. These also occurred to ARAF, in which the luciferase signal of N-luc-ARAF (Δaa1-202/BCT)-C-luc was further enhanced by introducing a canonical APE motif (A475P), suggesting that ARAF(Δaa1-245), ARAF (Δaa1-245/BCT), and ARAF (Δaa1-245/BCT/A475P) have a conformation of open monomer, partially open monomer, and close monomer respectively.

To further validate that deleting inhibitory N-terminus and replacing C-terminal tail as well as a canonic APE motif for ARAF will turn CRAF and ARAF into mature close monomers like BRAF, we mutated Cdc37/Hsp90-binding segments in CRAF and ARAF (G361A and F487L on CRAF and G322A and F448L on ARAF, which are identical to G469A and F595L of BRAF), and determined whether it activated mature close CRAF and ARAF monomers as it does in BRAF. Indeed, these mutations except F487L of CRAF triggered the activity of CRAF (Δaa1-245/BCT) and ARAF (Δaa1-245/BCT/A475P) respectively (Figure 6E-F).

### CRAF but not BRAF sustains a prolonged ERK signaling driven by EGF

**Figure 7:**
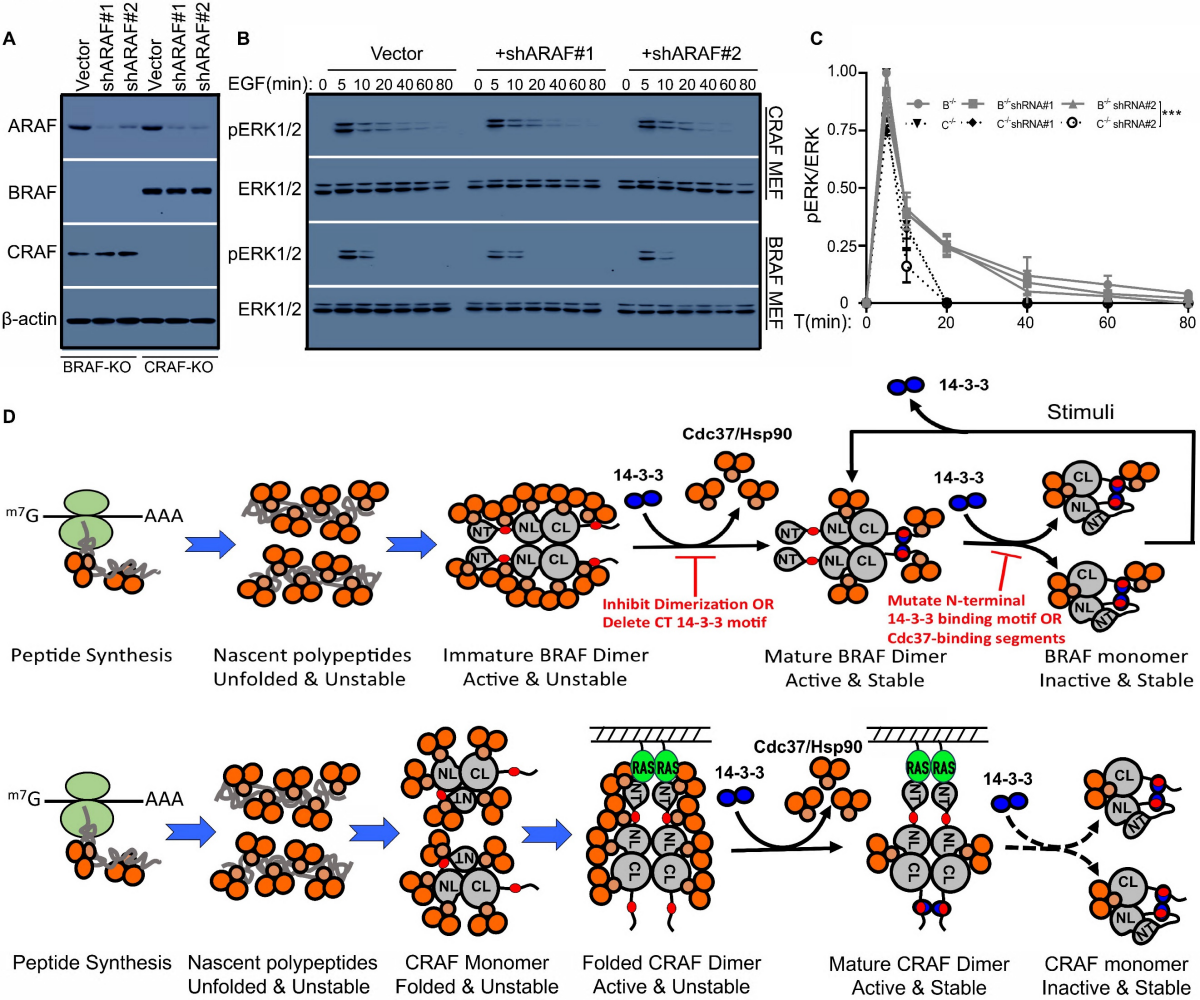
** RAF isoforms have different properties for transmitting ERK signaling downstream of RTK and RAS.** (A-C) In contrast to BRAF, CRAF sustained a prolonged ERK signaling upon EGF stimulation. The fibroblast cell lines that express only BRAF or CRAF were constructed by knocking down ARAF with lentiviral shRNAs in BRAF^-/-^ or CRAF^-/-^ fibroblasts, and expression of RAF isoforms was examined by immunoblots (A). EGF-induced phospho-ERK1/2 signaling in fibroblast cell lines from (A) was measured by immunoblot (B). Cells were stimulated with 100ng/ul EGF for indicated time and lysed for immunoblot. The ERK activity in (B) was quantified by using Image J (C). (D) A model for life cycles of RAF proteins. The nascent BRAF proteins are packed with Cdc37/Hsp90 chaperones, which facilitates BRAF folding into open monomers. Then these BRAF monomers dimerize and recruite 14-3-3 scaffolds to their C-terminus, which triggers a release of Cdc37/Hsp90 chaperones that recognize unfold/dynamic structures on BRAF proteins. However, some Cdc37/Hsp90 chaperones that recognize specific sequences (segments) of BRAF will be retained in BRAF complex and facilitate conversion of active open BRAF dimers into inactive close BRAF monomers with an arrangement of 14-3-3 binding modes. Upon stimulation, these inactive close BRAF monomers with intramolecular 14-3-3 association will be opened to re-dimerize with intermolecular 14-3-3 association, and eventually returned back to inactive close monomeric status with signaling decay. In contrast to BRAF, nascent CRAF proteins fold into close monomers with facilitation of CDc37/Hsp90 chaperones, in which their N-terminus dock on kinase domain and prevent CRAF dimerization as well as subsequent maturation. Hence CRAF proteins exist as immature close monomers in quiescent cells. Upon stimulation, active RAS proteins bind to N-terminus of CRAF proteins, which opens the close confirmation of CRAF proteins and promotes CRAF dimerization as well as C-terminal 14-3-3 association and Cd37/Hsp90 release. Since Cdc37/Hsp90 binding segment in C-terminal tail of CRAF is absent, active open CRAF dimers will be switched into inactive close monomers much more slowly than that for BRAF, which enables CRAF proteins to sustain a prolonged ERK signaling. As for ARAF, this regulatory process will be more complicate since it has a non-canonic APE motif that impairs dimerization. In addition, all RAF proteins with improper folding will be targeted by ubiquitin system for degradation if not packed by Cdc37/Hsp90 chaperones, which is not shown in this diagram. All images are representative of at least three independent experiments.

As shown in Figure 2, BRAF has three Cdc37/Hsp90-binding segments whose mutations promote BRAF dimerization and enable BRAF to sustain a prolonged ERK signaling. As for CRAF and ARAF, the Cdc37/Hsp90 binding segment in C-terminal tail is absent, and hence we next determined whether these two RAF isoforms were able to sustain a prolonged ERK signaling as those BRAF mutants. To do this, we firstly knocked down ARAF in BRAF- or CRAF-deficient fibroblasts, or CRAF in BRAF-deficient fibroblasts, or BRAF in CRAF-deficient fibroblasts, in order to obtain fibroblast cell lines that express a single RAF isoform. Although failed to produce any cell lines expressing only ARAF, we successfully generated cell lines expressing only BRAF or only CRAF (Figure 7A) with lentiviral shRNA transduction. We found that EGF-induced ERK signaling has much longer duration in cell lines expressing only CRAF than in those expressing only BRAF (Figure 7B-C), suggesting that CRAF really sustains a prolonged ERK signaling.

### A model for RAF maturation and cycling between active dimers and inactive monomers

In this study, we have investigated how Cdc37/Hsp90 chaperone and 14-3-3 scaffold proteins cooperatively regulate the stability and activity of RAF proteins. According to our data, we speculate that RAF proteins likely follow a life cycle as shown in Figure 7D: (1) For BRAF, nascent BRAF proteins initially associate with Cdc37/Hsp90 chaperones and fold into open monomers with their facilitation; then these monomeric BRAF proteins form open side-to-side dimers and recruit 14-3-3 scaffolds to their C-terminal tails, which stabilizes BRAF dimerization and triggers a release of Cdc37/Hsp90 chaperones that bound to unfold/dynamic structures of BRAF proteins; next Cdc37/Hsp90 chaperones that bound to specific segments of BRAF proteins switch BRAF proteins from open dimers into close monomers companying with a rearrangement of 14-3-3 binding modes from intermolecular to intramolecular associations. However, close BRAF monomers with intramolecular 14-3-3 association can be converted into open dimers with intermolecular 14-3-3 association upon stimulation. (2) For CRAF or ARAF, nascent RAF proteins fold into close monomers with facilitation from Cdc37/Hsp90 chaperones, in which their N-terminus fold back to dock on their kinase domain and hence prevent their side-to-side dimerization as well as subsequent maturation. Upon stimulation, active RAS-GTPs bind to their N-terminus and unfold their close conformation, which facilitates their dimerization as well as intermolecular 14-3-3 binding and Cdc37/Hsp90 release. In contrast to BRAF dimers, CRAF or ARAF dimers will be harder to switch into close monomers with intramolecular 14-3-3 association since they do not have a Cdc37/Hsp90-binding segment in their C-terminal tails, and hence sustain a prolonged ERK signaling upon activation. It should be noted that among three RAF isoforms, ARAF has a non-canonic AAE motif that reduces significantly ARAF dimer affinity 37, which makes its regulation more complicated. Additionally, immature RAF proteins, if not packed by Cdc37/Hsp90 chaperones, will be targeted by ubiquitin system for degradation.

## Discussion

The RAF family kinases have a well-defined role in cancer biology, and targeting RAF for treating cancers has achieved promising outcomes in clinical practice. However, there are still significant issues that need to be resolved in order to achieve a long-standing therapeutic efficacy as well as a wider coverage of cancers 53,54. This requires us to have better understanding of molecular mechanisms that regulate RAF function and how oncogenic mutations evade RAF regulatory machineries. In this study, we investigated how Cdc37/Hsp90 chaperone together with 14-3-3 scaffold regulates the maturation and activity of RAF kinases and how non-V600 mutations disrupt this regulation, which would have important implications for designing precise therapeutics against cancers harboring such mutations. Furthermore, since up-regulation of BRAF(V600E) as well as other RAF paralogs have been shown as one of the major causes that lead to RAFi resistance 55, our study would facilitate development of approaches for destabilizing RAF proteins and hence resolving this intractable problem.

Although all RAF isoforms have very similar molecular structures, our study indicates that their function is differentially regulated by Cdc37/Hsp90 chaperones and 14-3-3 scaffolds, which may arise from their different folding as well as dimer affinity. Specifically, nascent BRAF protein has an open conformation and high dimer affinity so that it can be eventually assembled into a mature close monomer through dimerization, 14-3-3 incorporation, Cdc37/Hsp90 release, and 14-3-3 rearrangement. In contrast, nascent CRAF and ARAF proteins have a close conformation in which their N-terminus fold back to block dimerization via C-terminal kinase domain, and also relatively low dimer affinity, which impairs their maturation and trap them in an immature close monomeric status. The maturation of CRAF and ARAF requires a facilitation of active RAS that binds to their N-terminus and open their close conformation for dimerization as well as subsequent 14-3-3 incorporation and Cdc37/Hsp90 release. Therefore, although all RAF isoforms exist as monomers in quiescent cells, BRAF stays in a mature status with less Cdc37/Hsp90 loading and more 14-3-3 association, whereas CRAF and ARAF are trapped in an immature status with higher Cdc37/Hsp90 loading and less 14-3-3 association. This may illustrate why BRAF is more stable than CRAF and ARAF and why BRAF is a dominant target of oncogenic mutations rather than CRAF and ARAF. In addition, absence of Cdc37/Hsp90-binding segment in C-terminal tail slows down active dimeric CRAF switching into inactive close monomers, and hence results in an oncogenic prolonged ERK signaling, suggesting that CRAF is more suitable than BRAF for sustaining oncogenic RAS mutation-driven tumorigenesis. Together with previous findings that RAF dimerization is critical for its activation, substrate phosphorylation and inhibitor resistance 33,41,56-60, here our study has shown once again that RAF dimerization is a determinant factor for its maturation.

Previous studies have shown that Cdc37/Hsp90 chaperone recognizes specific sequences (motifs) or unfold/dynamic structures on RAF proteins 12,14,17,40. Although we did not obtain direct evidence in this study, we speculate that these two types of associations of Cdc37/Hsp90 with RAF play different roles in regulating RAF function. Cdc37/Hsp90 chaperones engaged with unfold/dynamic structures on RAF proteins will be released upon 14-3-3 association, while those binding to specific sequences on RAF will always associate with RAF proteins and facilitate conversion of open dimeric RAF complex into close monomeric RAF complex. Most oncogenic non-V600 BRAF mutations occur on/around Cdc37/Hsp90-binding segments, which may disrupt sequence-dependent Cdc37/Hsp90 association and hence impair dimer-to-monomer switch of RAF proteins. Our data from both peptide affinity assay and co-immunoprecipitation assay support this notion. Furthermore, a recent study revealed an inverse correlation between Cdc37/Hsp90 chaperone affinity and homodimerization propensity for oncogenic BRAF β3-αC^del^ mutants 61, which is consistent with our opinion. However, why some mutants are constitutively active, but others have impaired kinase activity since all of them are trapped in dimeric status? This may arise from different roles of individual Cdc37/Hsp90-binding segments in the catalytic process of RAF proteins. The first Cdc37/Hsp90-binding segment localizes in Glycine-rich loop that involves in ATP binding. A bulky residue on this loop (i.e. G466V/E/R, G469E) may impair ATP loading and hence inhibit kinase activity, which will be examined in our future study. The second Cdc37/Hsp90-binding segment is DFG motif of RAF proteins that participates in ATP-binding (D594) and assembly of R-spine (F595 and G596), a typical hydrophobic architecture of active kinases 38,62. Mutations in DFG motif may disrupt ATP-binding or assembly of active conformation, and hence kill the catalytic activity of RAF proteins. As for F595L mutation in DFG motif, although a substitution of Phe with Leu slightly weakens R-spine, it significantly promotes BRAF dimerization through disrupting Cdc37/Hsp90 association, and hence elevates its kinase activity with net effect. However, a similar mutation F487L does not activate a mature close monomer of CRAF, CRAF(Δaa1-245/BCT), probably by virtue of its weaker R-spine. Additionally, a L597R mutation in this segment has been recently reported to disrupt Cdc37 association and improve dimerization 63. Overall, BRAF mutants with altered Cdc37/Hsp90-binding segments might directly or indirectly activate downstream signaling, both of which depend on their elevated dimer affinity.

With the reference to structures of RAF-Cdc37/Hsp90 and RAF-14-3-3 complexes 12-14,24, here we have revealed a framework for RAF maturation and its cycling between dimeric and monomeric status that regulated cooperatively by Cdc37/Hsp90 chaperones and 14-3-3 scaffolds. However, there are several important questions that remain resolved. Firstly, how does 14-3-3 engagement with dimeric RAF complex trigger a release of Cdc37/Hsp90 chaperones that sense unfold/dynamic structures on RAF? Secondly, how does Cdc37/Hsp90 chaperones that sense specific sequences of RAF facilitate a dimer-to-monomer switch of BRAF complex as well as 14-3-3 scaffold rearrangement? Thirdly, although our study has suggested that CRAF and ARAF have quite different life cycles in contrast BRAF, how their function is precisely regulated by Cdc37/Hsp90 chaperones and 14-3-3 scaffolds under physiological conditions, and how does its disruption cause diseases? Addressing these questions will not only improve our understanding of RAF regulatory mechanism, but also accelerate anti-RAF therapeutic development.

## Methods and Materials

### Antibodies and other biological reagents

Antibodies used in this study include: anti-phospho-ERK1/2 (#4370), anti-phospho-CRAF (Ser259) (#9421), anti-CRAF (#9422), anti-Cdc37 (#4793), and anti-14-3-3 (#8312) anti-ARAF (#4432) (Cell Signaling Technology); anti-HA (MAB6875, Novus Biologicals), anti-FLAG (F3165, Sigma-Aldrich); anti-β-actin (#66009, Proteintech); anti-Hsp90 (#A0365, ABclonal); anti-ERK1/2 (sc-514302) and anti-BRAF (sc-5284) (Santa Cruz Biotechnology); anti-phospho-CRAF (Ser621) (44504G, Invitrogen); anti-Ki67 (ab16667, Abcam); and HRP-labeled secondary antibodies (#31460, #31430, Invitrogen). plx4720 (T2473) and plx8394 (T3579) were purchased from TargetMol Inc., while recombinant EGF from Bio-Rad Laboratories (#PHP030A). All other biochemicals were obtained from sigma-tau (RRID:SCR_000488).

Wild-type, BRAF^-/-^ and CRAF^-/-^ fibroblasts were gifts from Professor Manuela Baccarini at University of Vienna, Austria 64,65. 293T cell lines were purchased from ATCC.

Plasmids encoding BRAF, CRAF, ARAF, and their mutants as well as Cdc37 were constructed by using Gibson assembly. Lentiviral shRNA vectors for knocking down ARAF were constructed in previous study 66. pCDNA3.1(+) vector (Invitrogen) was used for transient expression; and pMSCV retroviral vectors (Clontech) for stable expression.

### Cell culture, transfection, and transduction

All cell lines were maintained in DMEM medium with 10% FBS (Hyclone). Cell transfections were carried out by using the lipofectamine 2000 transfection reagent (Invitrogen). To reconstitute BRAF^-/-^ or CRAF^-/-^ fibroblasts, viruses encoding BRAF, CRAF, or their mutants were prepared and applied to infect cells according to our previous studies 37,66. GFP^+^ infected cells that express the equal level of RAF proteins were sorted out by flow cytometer for subsequent experiments since both RAF and GFP were generated from a RAF-IRES-GFP cassette in retroviral vectors.

### Peptide synthesis and Cdc37 affinity assay

The peptides that represent for different Cdc37 binding sites of BRAF or oncogenic mutants were synthesized on a cellulose-balanine-membrane as spots (JPT, Berlin, Germany). The membrane was rinsed with methanol, washed with TBS-T and blocked for 2 hours using 5% BSA. The saturated membrane was incubated with whole cell lysates prepared from 293T transfectants of Cdc37 for overnight in cold room. After washing gently with PBS-T, the membrane was incubated in cold room with anti-Cdc37 antibody for 4~6 hours and then with HRP-conjugated secondary antibody for 2 hours. Cdc37 proteins associated with peptide spots on the washed membrane were visualized using the ECL system (Bio-Rad) and quantified using the software ImageJ, RRID:SCR_003070.

### Immunoprecipitation and western blotting

Immunoprecipitations were performed as described previously 37,44,50,66. Briefly, 293T transfectants were lysed on ice with RIPA buffer (50 mM Tris, 150 mM NaCl, 1 mM EDTA, pH7.2) containing 0.2% NP-40 and inhibitors for proteases, kinases and phosphatases, and used to prepare whole-cell lysates. Then whole-cell lysates were mixed with anti-FLAG beads (A2220) (Sigma), rotated in cold room for 120 min, and quickly washed three times with RIPA buffer. The immunoprecipitants were mixed with 2X SDS sample buffer, and run on SDS-PAGE after boiling at 85 ^o^C for 2 min. Proteins on SDS-PAGE were transferred onto nitrocellulose membrane and the immunoblottings were carried out as described before 44,45. It should be noted that if used only for immunoblotting, whole-cell lysates could be prepared with RIPA buffer containing 1% NP-40, otherwise RIPA buffer with 0.2% NP-40 should be used since high-concentration NP-40 dissociated RAF complexes in whole cell lysates.

### Complementary split luciferase reporter assay

293T transfectants that express N-luc-BRAF-C-luc, N-luc-CRAF-C-luc, N-luc-ARAF-C-luc proteins or their mutants respectively were plated in 24-well Krystal black image plates at a seeding density of 2x10^5^ cells per well. Twenty-four hours later, d-luciferin (0.2 mg/ml) was added to the culture, and the incubation was allowed for 30 min before the luciferase signals were measured by using Promega GloMax-Multi Detection System.

### Animal studies

For xenograft experiments, female NOD/SCID mice (6~8 weeks) were subcutaneously injected with 5x10^6^ cells per mice in 1:1 matrigel (Corning). Tumor volumes were monitored by calipers twice a week and calculated using the formula: volume= (width)^2^ x length/2. plx4720 and plx8394 was administered orally (200 mg/kg) twice a day when tumors reached an average volume of 50-60 mm^3^. At the experiment endpoint, mice were euthanized, and tumors were harvested for *ex vivo* analysis and subsequent histology. All operations were approved by the Animal Ethics Committee of NCCS.

### Immunohistochemistry staining

Tumors were fixed in 10% buffered formalin overnight and embedded according to standard procedures. Tumor sections were cut to 5 µm thickness, mounted on glass slides, and air-dried at room temperature. After antigen retrieval, tumor sections were stained with antibodies and then with hematoxylin. Images of tumor sections were taken with a bright light microscope at X10.

### Statistical analysis

All statistical analysis in this study was performed using GraphPad Prism, RRID:SCR_002798 (GraphPad Software, CA, USA). Statistical significance was determined by two-tailed Student's *t*-test in animal studies and error bars represent s.d. to show variance between samples in each group, or by one-sample *t*-test in other experiments and error bars represent s.d. to show variance between independent experiments.

## Supplementary Material

Supplementary figures.
