# Supplementary material for: Oncogenic non-V600 mutations evade the regulatory machinery of RAF including the Cdc37/Hsp90 chaperone and the 14-3-3 scaffold
Source: Theranostics. 2025 Jan 6;15(5):2035–51. doi: 10.7150/thno.103958 (PMC11780520; doi:10.7150/thno.103958)
Supplement: Supplementary file 1 — Supplementary figures. [file thnov15p2035s1.pdf]

Supplementary Information

Figure S1

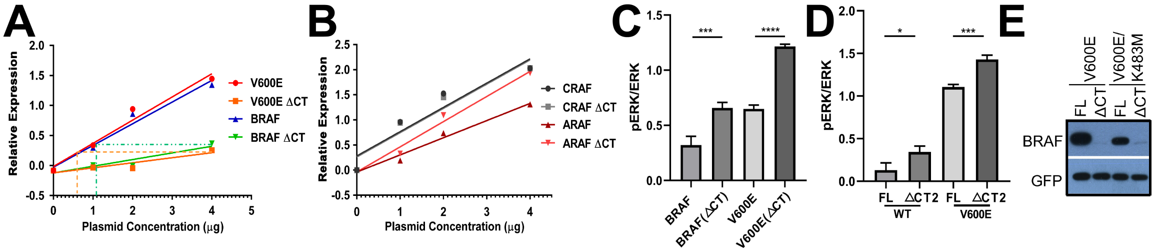

Figure S1. Deleting the carboxyl-terminus of BRAF but not CRAF or ARAF destabilizes protein and elevates its activity. (A) The expression quantification of BRAF and its V600E mutant with or without carboxyl-terminal truncation in Figure 1A. (B) The expression quantification of CRAF and ARAF with or without carboxyl-terminal truncation in Figure 1B. (C) The activity quantification of BRAF and its V600E mutant with or without carboxyl-terminal truncation in Figure 1C. *** p<0.001; **** p<0.0001. (D) The activity quantification of BRAF and V600E mutant with or without truncation of carboxyl-terminal Cdc37/Hsp90-binding segment in Figure 1F. * p<0.05; *** p<0.001. (E) The kinase activity of BRAF(V600E) did not involve in its stability regulation by carboxyl-terminus. Plasmids encoding BRAF(V600E), BRAF(V600E/K483M), or their counterparts with carboxyl-terminal truncation in a BRAF-IRES-GFP cassette were transfected into 293T cells, and the expression of target proteins in 293T transfectants was measured at 24 hours after transfection by immunoblot with GFP as an internal control.

Figure S2

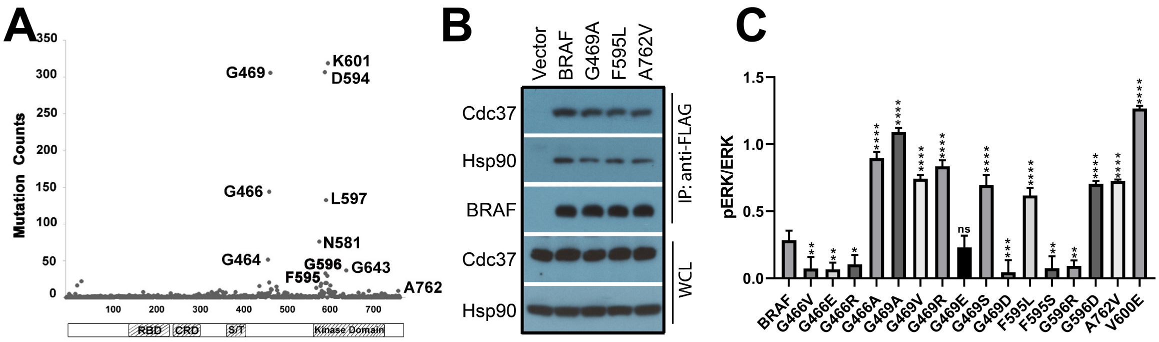

Figure S2. Oncogenic non-V600 mutations target Cdc37/Hsp90-binding segments of BRAF. (A) A statistic analysis of non-V600 BRAF mutations in cancer genomes. The data was extracted from the COSMIC database. As shown in this panel, most prominent non-V600 mutations occurred on or close to the Cdc37/Hsp90-binding segments ($_{466}$GSFG$_{469}$, $_{593}$GDFG$_{596}$, and $_{758}$GGYGAF$_{763}$). (B) Three representative mutations on Cdc37/Hsp90-binding segments slightly inhibited Cdc37/Hsp90 chaperone association. BRAF or its mutants were expressed in 293T cells, pulled down from whole cell lysates by immunoprecipitation, and Cdc37/Hsp90 in immunoprecipitants were examined by immunoblots. (C) The quantification of ERK signaling induced by non-V600 mutants in 293T cells in Figure 2C. * p<0.05; ** p<0.01; *** p<0.001; **** p<0.0001.

Figure S3

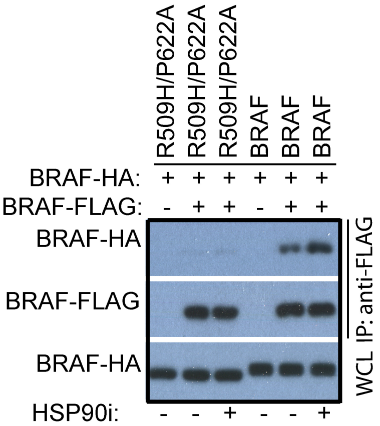

Figure S3. A transient treatment of 293T transfectants with Hsp90 inhibitor improves BRAF dimerization. The 293T transfectants expressing BRAF with either FLAG or HA tag or both were treated with 0.5 uM 17-AAG for 90 min, and then lyzed with RIPA buffer containing 0.2% NF-40 for co-immunoprecipitation assay as in Figure 3D.

Suppl. Figure 4

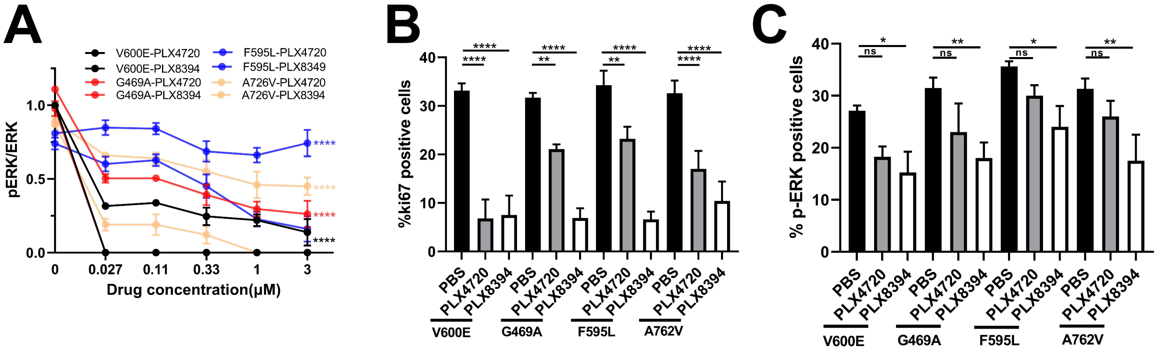

Figure S4. BRAF mutants with altered Cdc37/Hsp90 binding segments are resistant to type 1.5 RAF inhibitor plx4720 but not to RAF dimer breaker plx8394 in vitro and in vivo. (A) The quantification of ERK signaling in Figure 3A. **** p<0.0001. (B) The quantification of Ki-67 staining in Figure 3E. ** p<0.01; **** p<0.0001. (C) The quantification of phospho-ERK1/2 staining in Figure 3F. n.s. no significance; * p<0.05; ** p<0.01.

Figure S5

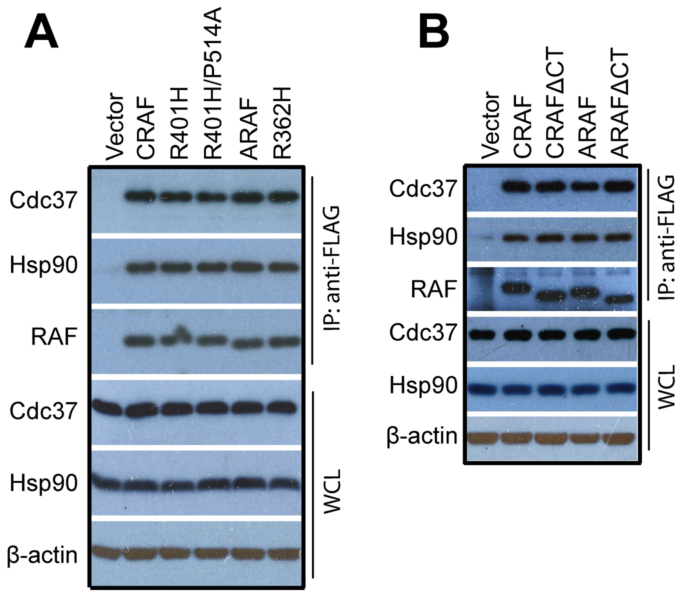

Figure S5. Deleting the carboxyl-terminus of CRAF and ARAF or disrupting their dimer interface does not alter their Cdc37/Hsp90 engagement. (A-B) CRAF, ARAF and their mutants were expressed in 293T cells, and pulled down by immunoprecipitation. RAF, Cdc37 and Hsp90 in immunoprecipitants were detected by immunoblots.

Figure S6

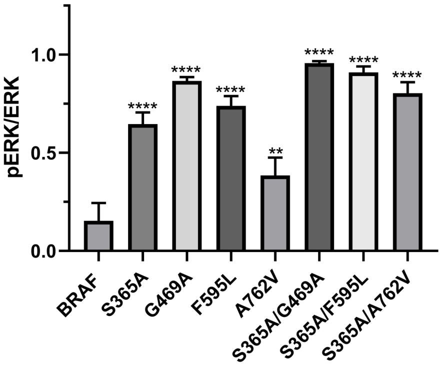

Figure S6. The quantification of ERK signaling induced by BRAF mutants in 293T cells in Figure 5C. ** p<0.01; **** p<0.0001.

Figure S7

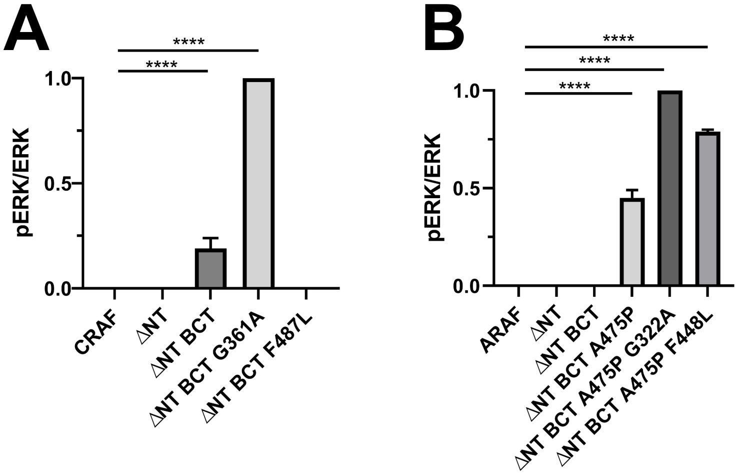

Figure S7. The maturation and activity of CRAF and ARAF proteins is regulated cooperatively by Cdc37/Hsp90 chaperones and 14-3-3 scaffolds via a manner similar to that for BRAF. (A) The quantification of ERK signaling induced by CRAF mutants in 293T cells in Figure 6E. **** $p<0.0001$. (B) The quantification of ERK signaling induced by ARAF mutants in 293T cells in Figure 6F. **** $p<0.0001$.
